# Microbiome as a predictive biomarker in locally advanced rectal cancer

**DOI:** 10.20517/mrr.2024.85

**Published:** 2025-03-24

**Authors:** Núria Mulet Margalef, Berta Martín Abad, Eva Martínez-Balibrea, Jose Luís Manzano Mozo, Alessandra Borgognone, Mireia Obón-Santacana

**Affiliations:** ^1^Medical Oncology Department, Catalan Institute of Oncology (ICO), Badalona 08916, Spain.; ^2^Germans Trias i Pujol Institute (IGTP), Translational Program in Cancer Research (CARE), Badalona 08916, Spain.; ^3^ProCURE Program, Catalan Institute of Oncology, Badalona 08916, Spain.; ^4^IrsiCaixa, Hospital Universitari Germans Trias i Pujol, Badalona 08916, Spain.; ^5^Unit of Biomarkers and Suceptibility (UBS), Oncology Data Analytics Program (ODAP), Catalan Institute of Oncology (ICO), L’Hospitalet del Llobregat, Barcelona 08908, Spain.; ^6^ONCOBELL Program, Bellvitge Biomedical Research Institute (IDIBELL), L’Hospitalet de Llobregat, Barcelona 08908, Spain.; ^7^Consortium for Biomedical Research in Epidemiology and Public Health (CIBERESP), Madrid 28029, Spain.; ^#^Authors contributed equally.

**Keywords:** Microbiome, locally advanced rectal cancer, epigenomics, exposome, *Fusobacterium nucleatum*

## Abstract

The incidence of locally advanced rectal cancer (LARC) among young people is rising alarmingly. In recent years, new protocols have been introduced for the management of LARC, some of which are associated with the risk of significant toxicity. Despite these advancements, robust predictive biomarkers for LARC have yet to be established. The microbiome has emerged as a potential biomarker due to its interaction with tumor multiomics. This article provides a critical overview of the current evidence on the microbiome and LARC, including its relationship with the immune system and epigenomics, and also highlights both the current limitations and future perspectives in the field.

Colorectal cancer (CRC) is one of the three most prevalent cancers worldwide and a leading cause of cancer-related deaths^[1]^. From a pathogenesis perspective, Fearon and Vogelstein proposed a model in which multiple genomic aberrations accumulate progressively, leading to the malignant transformation from healthy mucosa to invasive carcinoma^[2]^. However, in recent years, growing evidence has highlighted the significance of the exposome in CRC development and outcomes^[3]^. Diet is among the most studied factors.

An anti-inflammatory diet, for instance, promotes the production of short-chain fatty acids that help maintain gut barrier integrity^[4]^. This dietary pattern has been associated with better outcomes in patients with stage III CRC featuring high-risk characteristics^[5]^. In contrast, a Western diet has been linked to a higher incidence of CRC, particularly in individuals with the presence of *Fusobacterium nucleatum* (*Fn*)^[6]^ and pks+ *Escherichia coli*^[7]^. However, it is essential to consider the significant interindividual variability in CRC risk and evolution once diagnosed^[8]^. Beyond diet, other factors - such as physical activity^[9]^, air pollution^[10]^, microplastic intake^[11]^, and disruptions in circadian rhythms^[12]^ - are emerging as important contributors to CRC development. Given this complexity, molecular pathology epidemiology offers a framework to better understand the interactions between the external exposome and internal factors (including the microbiome). This framework also integrates multi-omics data (such as genomics, transcriptomics, immune contexture, and metabolomics) to provide a comprehensive view of CRC in individual patients^[13]^. In the near future, this approach should be incorporated into multimodal management strategies for CRC.

Rectal cancer accounts for approximately one-third of CRC cases, and it is one of the main tumor sites in patients under 50 years of age^[1,14]^. These patients belong to the entity early-onset CRC (EOCRC), and its incidence has been increasing globally^[1,14]^. Since 23% of rectal cancer cases are diagnosed in young people^[15]^, EOCRC represents a significant health concern from medical, social, emotional, and occupational perspectives. It also poses a major challenge for the scientific community, as the underlying causes of EOCRC remain poorly understood^[15]^. It has been hypothesized that individuals born after 1960 may be part of a potential cohort effect, with increased exposure to various risk factors, such as “Western” dietary patterns, rising childhood and adolescent obesity, reduced physical activity, antibiotic use, and increased artificial feeding^[14]^. However, further research is required to validate these hypotheses. In addition to the EOCRC issue, rectal cancer - particularly locally advanced rectal cancer (LARC) - faces another challenge related to its therapeutic approach. In recent years, the classical preoperative protocols for LARC, which combine neoadjuvant radiotherapy (RT) with chemotherapy (CT) using fluoropyrimidines, have given way to total neoadjuvant treatment (TNT) regimens. The goal of TNT is to improve the rate of complete clinical responses (cCR)^[16]^. TNT regimens typically include CT-RT, followed by or preceded by polychemotherapy (mainly oxaliplatin and fluoropyrimidines), before standard surgery with total mesorectal excision^[16]^. However, these regimens may cause disabling adverse events, such as severe neurotoxicity, affecting up to 25% of patients^[17]^. Among patients who achieve a cCR - estimated at 16%-30%^[18]^ - some may be eligible for Watch and Wait protocols at specialized centers, potentially avoiding surgical morbidity, including fecal and urinary incontinence, and sexual dysfunction^[16]^. However, the long-term prognostic impact for patients who experience tumor regrowth remains unclear^[19]^.

One of the key challenges in LARC is the identification of predictive biomarkers for response and toxicity to neoadjuvant treatments. Currently, the pathological complete response and its correlation with improved survival^[20]^, as well as the MSI-H/dMMR phenotype in less than 10% of patients and the associated benefit from immunotherapy^[21]^, are the most relevant clinical examples. Recently, the microbiome has emerged as a potential biomarker for LARC.

Two meta-analyses published in 2019 found that the fecal microbiome in CRC patients is richer compared to non-CRC individuals, with a higher abundance of oral species, but with no differences in diversity. Functional analysis revealed an association between putrefaction, fermentation, and gluconeogenesis pathways with CRC^[22,23]^. Additionally, several species, such as *Parvimonas spp.*, *Fn*, and *Peptostreptococcus stomatis*, were overrepresented in CRC samples compared to controls^[22,23]^. In LARC, multiple studies have described differences in microbiome patterns between pre- and post-neoadjuvant treatment in tumor or fecal samples, and these changes are preliminarily correlated with treatment benefits [Table 1].

**Table 1:** Examples of studies that have preliminarily identified associations between the presence of specific bacterial species and the outcomes of LARC patients undergoing neoadjuvant therapy

**Authors**	***n***	**Sample**	**Technique**	**Negative association**	**Positive association**
Teng *et al.*^[54]^	353	Fecal	16S rRNA	*Bacteroides vulgatus*	
White *et al.*^[57]^	107	Tumor and adjacent normal tissue	Metagenomic analysis		*Fn*, *Bacteroides dorei*, *Ruminococcus bromii*
Yi *et al.*^[58]^	84	Fecal	16S rRNA	*Coriobacteriaceae*, *Fusobacterium*	*Roseburia*, *Dorea*, *Anaerostipes*
Huang *et al.*^[53]^	73	Tumor tissue	Metagenomic analysis	*Streptococcus equinus*, *Schaalia odontolytica*, *Clostridium hylemonae*, *Blautia producta*, *Pseudomonas azotoformans*	
Takenaka *et al.*^[59]^	44	Tumor tissue	16S rRNA	*Paraprevotella*, *Enhydrobacter*	*Hungatella*, *Flavonifractor*, *Methanosphaera*

LARC: Locally advanced rectal cancer; *Fn*: *Fusobacterium nucleatum.*

Among the various species, *Fn*, an anaerobic Gram-negative bacillus, is one of the most studied. A seminal study involving 143 LARC patients found that the persistence of *Fn* in surgical specimens, determined by RNA *in situ* hybridization (RNA-ISH) after CT/RT neoadjuvant treatment, was associated with a higher risk of local or distant recurrence [hazard ratio (HR) = 7.5, 95%CI: 3.0-19; *P* < 0.001]^[24]^. Interestingly, the presence of *Fn* in diagnostic samples did not show the same correlation, nor did it correlate with the degree of response to neoadjuvant treatment. However, a statistically significant increase in the density of CD8^+^ cells was observed in patients who had no *Fn* in either pre- or post-neoadjuvant samples, or in those with *Fn* present only in pre-treatment samples, compared to patients with *Fn* present in both samples. These results suggest that *Fn* may be associated with reduced activation of the cytotoxic immune response, in line with preclinical data showing that *Fn* uses its virulence proteins to bind negative regulators of the immune cell cycle, such as TIGIT^[25]^ and CEACAM1^[26]^, leading to poor CD4^+^/CD8^+^ T-cell infiltration^[27]^. Contrary to what might be expected based on these properties, *Fn* has been preliminarily proposed as a favorable biomarker for immunotherapy benefit in MSS CRC. Preclinical data suggest that *Fn* may enhance the cytotoxic effect of CD8^+^ T cells through the generation of butyric acid, which mediates epigenomic events that lead to reduced PD-1 expression^[28]^.

Despite promising results that are still pending validation in further studies, several caveats should be considered. Firstly, the prevalence of *Fn* in LARC is not well established. According to one of the largest series, which included more than 1,000 patients, the proportion of rectal cancers with high levels of *Fn* - detected by polymerase chain reaction (PCR) - is 2.5%^[29]^. However, this proportion likely reflects substantial inter- and intraindividual variability due to exposome-related factors such as geographic region and diet.

Secondly, there is no consensus on the best methodology for identifying this bacterium and its associated microbial communities, including sample processing, bioinformatics tools, and workflows. Moreover, the degree of concordance between microbiome patterns in fecal and tumor tissue remains largely unexplored, with existing data showing inconsistencies^[30,31]^. This issue is particularly relevant for *Fn*, as its representation in tumor tissue is higher than in fecal samples when 16S rRNA sequencing is used^[32]^.

Thirdly, a comprehensive understanding of the oncogenic properties of *Fn* is necessary to fully determine whether its presence in LARC is a consequence of *Fn* itself, which could lead to new therapeutic strategies, or whether it results from tumor evolution that promotes *Fn* colonization. Preliminary data suggest that *Fn* exhibits pro-tumoral traits, as it promotes gut tumorigenesis in APC^Min/+^ mice through the activation of oncogenic pathways and modulation of the tumor microenvironment. *Fn* binds to Toll-like receptor 4, triggering MAPK pathway activation via miR-21^[33]^ in CRC cell lines. It also activates the Wnt pathway by binding E-cadherin on CRC cells through FadA, one of its virulence proteins^[34,35]^.

Finally, given the complexity of the human microbiome and its interactions with the host’s (epi)genetics, the transcriptional processes, and the immune system, it is currently difficult to propose a single species as a robust biomarker for LARC.

In parallel with LARC, microbiome analysis has also emerged as a potential biomarker for response and toxicity to immune checkpoint inhibitors (ICIs) in oncology. This opens the door to lessons learned from other cancer types that could be applied to LARC. For example, *Akkermansia muciniphila* has been preliminarily associated with better outcomes with ICIs, especially in non-small cell lung cancer^[36]^. The modulation of antitumor immune response by the microbiota occurs through various mechanisms: production of immunomodulatory metabolites by bacteria^[37,38]^, bacterial migration from the gut to other organs^[39]^, antigen mimicry^[40]^, and direct modulation of the tumor microenvironment via immune checkpoint expression^[41]^, lymphocyte trafficking molecules^[42]^, or the production of chemokines like CXCL13^[43]^. Taking into account this network, bioinformatics approaches that consider not only microbial groups but also their interplay with the human host acting as a functional ecosystem, may offer more accurate predictions for immunotherapy outcomes^[36]^. This approach should be considered for LARC as more information becomes available, especially from mechanistic studies evaluating the immunomodulatory effects of RT and oxaliplatin-based CT.

In addition to immune responses, the analysis of epigenomic events is also highly relevant in LARC, given the rising incidence among young people^[2]^. Epigenomic analysis, using various approaches in tumor tissue, could provide valuable insights. One such approach involves the determination of methylation markers in specific genes, such as the hypomethylation of transcription factor AP-2 epsilon (TFAP2E)^[44]^ and the hypermethylation of O-6-methylguanine-DNA methyltransferase (MGMT)^[45]^, both of which are associated with a better response to neoadjuvant treatment. Additionally, baseline hypomethylation of long interspersed element-1 (LINE-1), which is linked to genomic instability, has been preliminarily associated with poorer survival and a higher risk of recurrence in LARC^[46]^, as well as with physical inactivity, smoking, high BMI, and pesticide exposure^[47]^. Other approaches include determining the methylator phenotype (CIMP-H), although results have been inconclusive, or examining genome-wide methylation markers, which may prove more useful. For example, a preliminary study with 53 LARC patients treated with RT and 5FU-based CT showed that treatment could alter methylation patterns in tissue, and that low initial methylation levels were associated with a higher probability of treatment response^[48]^. Furthermore, the use of CpG island methylation arrays revealed that methylation patterns in regions near transcriptional regulatory zones of genes regulated by enhancer of zeste homolog 2 (EZH2) could discriminate the prognosis of LARC patients treated with neoadjuvant therapy^[49]^.

Moreover, a link between epigenomics and the microbiome has been preliminarily described. A study in murine models showed that crypt cells in germ-containing models exhibited hypomethylation of elements regulating inflammatory processes compared to germ-free models. This was attributed to increased expression of ten-eleven-translocation 3 (TET3), an enzyme involved in DNA demethylation^[50]^. Similarly, it has been suggested that certain metabolites produced by gut bacteria, such as folate (produced by *Bifidobacterium* and *Lactobacillus*) and short-chain fatty acids like butyrate (mostly produced by Firmicutes), can influence DNA methylation^[51,52]^. Additionally, the presence of butyrate-producing bacteria in tissue has been associated with resistance to CT/RT^[53]^, as has nucleotide biosynthesis mediated by *Bacteroides vulgatus*^[54]^.

Finally, beyond its neoplastic effects, the microbiome has been preliminarily linked to surgical complications in LARC, such as low anterior resection syndrome^[55]^, and even with depressive symptoms and sleep disturbances^[56]^, suggesting the relevance of the gut-brain axis.

In conclusion, the microbiome holds promise as a biomarker in LARC. However, further collaborative efforts are needed to clarify its role in tumor carcinogenesis and standardize the methodology of analysis. Validation studies in patients should also consider the evolving exposome and other “omics” fields, such as epigenomics, to provide a comprehensive understanding of this disease, which notably affects young people.

## DECLARATIONS

### Copyright

© The Author(s) 2025.
